# Common *UCP2* variants contribute to serum urate concentrations and the risk of hyperuricemia

**DOI:** 10.1038/srep27279

**Published:** 2016-06-08

**Authors:** Luyu Yang, Zheng Dong, Jingru zhou, Yanyun Ma, Weilin Pu, Dongbao Zhao, Hongjun He, Hengdong Ji, Yajun Yang, Xiaofeng Wang, Xia Xu, Yafei Pang, Hejian Zou, Li Jin, Chengde Yang, Jiucun Wang

**Affiliations:** 1 State Key Laboratory of Genetic Engineering and Ministry of Education Key Laboratory of Contemporary Anthropology, Collaborative Innovation Center for Genetics and Development, School of Life Sciences, Fudan University, Shanghai, China; 2 Division of Rheumatology and Immunology, Changhai Hospital, Shanghai, China; 3 Division of Rheumatology, Taixing People’s Hospital, Jiangsu Province, China; 4 Division of Rheumatology, Taizhou People’s Hospital, Jiangsu Province, China; 5 Fudan-Taizhou Institute of Health Sciences, Taizhou, Jiangsu Province, China; 6 Division of Rheumatology, Huashan Hospital, Fudan University, Shanghai, China; 7 Institute of Rheumatology, Immunology and Allergy, Fudan University, Shanghai, China; 8 Division of Rheumatology, Ruijin Hospital, Shanghai Jiaotong University School of Medicine, Shanghai, China

## Abstract

Elevated serum urate, which is regulated at multiple levels including genetic variants, is a risk factor for gout and other metabolic diseases. This study aimed to investigate the association between *UCP2* variants and serum urate as well as hyperuricemia in a Chinese population. In total, 4332 individuals were genotyped for two common *UCP2* variants, −866G/A and Ala55Val. These loci were not associated either serum urate level or with a risk of hyperuricemia in the total group of subjects. However, in females, −866G/A and Ala55Val were associated with a lower serum urate (*P* = 0.006 and 0.014, seperately) and played a protective role against hyperuricemia (OR = 0.80, *P* = 0.018; OR = 0.79, *P* = 0.016). These associations were not observed in the males. After further stratification, the two loci were associated with serum urate in overweight, but not underweight females. The haplotype A-T (−866G/A-Ala55Val) was a protective factor for hyperuricemia in the female subgroup (OR = 0.80, *P* = 0.017). This present study identified a novel gene, *UCP2*, that influences the serum urate concentration and the risk of hyperuricemia, and the degree of association varies with gender and BMI levels.

Uric acid is the final product of purine oxidation in humans. Elevated serum urate, or hyperuricemia, has long been recognized as an independent risk factor for gout^1^,^2^. There is a renewed interest in hyperuricemia and its association with a number of other clinical disorders including hypertension, atherosclerosis, cardiovascular disease, chronic kidney diseases, and abdominal obesity, glucose intolerance, insulin resistance, and dyslipidemia, which are often subsumed under the term “metabolic syndrome”^3^.

Serum urate is balanced between uric acid production in the liver and its disposal via the kidney and gut^4^. The occurrence of hyperuricemia could be caused by disruptions in any part of this metabolic process. Both genetic and environmental factors, such as gender and body mass index (BMI), have a strong effect on the risk of hyperuricemia^3^. Among those factors, the attribution of genetic factors is estimated to be as high as 73%^5^. Recent genome-wide association studies (GWAS) have identified 28 loci associated with serum urate concentration^6^. However, only approximately 7% of the variation in serum urate concentration could be explained by those reported loci, suggesting the missing heritability remained to be explored^6^.

Human uncoupling proteins (UCPs) are mitochondrial transporters present in the inner membrane of mitochondria^7^. UCPs are capable of uncoupling ATP production from mitochondrial respiration by causing proton leak and preventing mitochondrial hyperpolarization and the formation of reactive oxygen species (ROS)^8^. Among the five identified UCPs, UCP2 is widely expressed in almost all mammalian tissues including white adipose tissue, liver, kidney, pancreatic islets, macrophages and retinal endothelial cells, indicating its involvement in a variety of physiologic or pathologic events^9^,^10^,^11^,^12^. Two of the most common polymorphisms of this gene, −866G/A (rs659366) in the promoter and Ala55Val (rs660339) in codon 55, were identified as being associated with different phenotypes^7^,^12^, including obesity, insulin resistance, type 2 diabetes mellitus (T2D), low-density lipoprotein (LDL) particle size, coronary incidence and other metabolic disorders^9^,^10^,^13^,^14^,^15^,^16^,^17^,^18^,^19^,^20^,^21^.

Given the involvement of *UCP2* and hyperuricemia in a variety of metabolic disorders, we selected the two common loci −866G/A and Ala55Val to explore the association between genetic *UCP2* variants and hyperuricemia in a Chinese population, offering a new diagnostic or therapeutic target for hyperuricemia.

## Results

### There was no association between SNPs and serum urate

The two loci were proven in Hardy-Weinberg equilibrium (−866G/A: *P* = 0.990; Ala55Val: *P* = 0.690). For −866G/A, AA, AG, and GG genotypes accounted for 21.6%, 49.9%, and 28.6% of hyperuricemic patients, respectively; in healthy controls, the distribution was 21.2%, 49.6%, and 29.3%, respectively. As shown in Table 1, the −866G/A polymorphism was not found to be associated with serum urate (AA/GG: Beta = −0.008, *P* = 0.644; AG/GG: Beta = −0.012, *P* = 0.474) or with the risk of hyperuricemia (AA/GG: OR = 1.05, *P* = 0.603; AG/GG: OR = 1.03, *P* = 0.667). For Ala55Val, the TT, TC, and CC genotype distribution was 21.5%, 50.5% and 28.0% in hyperuricemic patients, respectively, and the distribution was 21.5%, 49.8% and 28.6% in healthy controls, respectively. No association was observed between Ala55Val polymorphism and serum urate (TT/CC: Beta = −0.013, *P* = 0.460; TC/CC: Beta = −0.017, *P* = 0.324). There was no difference in the distribution of the genotypes or alleles among hyperuricemic patients and healthy controls (TT/CC: OR = 1.02, *P* = 0.824; TC/CC: OR = 1.04, *P* = 0.652). Therefore, no statistically solid evidence supported the genetic effect of −866G/A and Ala55Val on serum urate or the risk of hyperuricemia in the total group of subjects.

### *UCP2* variants were associated with serum urate and hyperuricemia in female subgroups

As shown in Table 1, we stratified all subjects into male and female subgroups to further explore the gender-related genetic effects of the two polymorphisms. In the male subgroups, there were no significant associations between the two loci and serum urate or the risk of hyperuricemia (all *P *> 0.025). However, some nominal significant associations were found between −866G/A and the hyperuricemia risk (genotype AA: OR = 1.26, *P* = 0.038; allele A: OR = 1.12, *P* = 0.035), indicating a possible risky effect of the −866G/A variant on hyperuricemia incidence in males.

A significant association was found between SNPs and serum urate and hyperuricemia in the female subgroups. The −866G/A genotypes were associated with a lower serum urate (AA/GG: Beta = −0.078, *P* = 0.015; AG/GG: Beta = −0.104, *P* = 0.001) and a decreased risk of hyperuricemia (AG/GG: OR = 0.71, *P* = 0.025). The subjects carrying allele A had a lower serum urate and a decreased risk of hyperuricemia (A/G: Beta = −0.054, *P* = 0.006; OR = 0.80, *P* = 0.018). For Ala55Val, genotype TT carriers showed a lower serum urate (TT/CC: Beta = −0.075, *P* = 0.022) and a decreased risk of hyperuricemia (TT/CC: OR = 0.64, *P* = 0.020). Genotype TC carriers only had a lower serum urate (TC/CC: Beta = −0.082, *P* = 0.012) but no decreased risk of hyperuricemia (TC/CC: OR = 0.77, *P* = 0.093). Allele T was associated with a lower serum urate (T/C: Beta = −0.049, *P* = 0.016) and a decreased risk of hyperuricemia (T/C: OR = 0.79, *P* = 0.016).

### Further analysis of association in females with different BMI levels

Further analysis was performed regarding the genetic effect of *UCP2* variants on serum urate and the risk of hyperuricemia among females with different BMI levels (Table 2). The majority of the females enrolled were stratified into normal- or overweight group (Table 2). In the underweight subgroup, whose sample size was limited after stratification, no significant association was observed between the two loci and serum urate or hyperuricemia risk (all *P *> 0.025, Table 2). In the normal weight subgroup, −866G/A genotype AA + AG carriers were associated with a lower serum urate (AA + AG/GG: Beta = −0.095, *P* = 0.022) but not with a decreased risk of hyperuricemia (AA + AG/GG: OR = 0.65, *P* = 0.076). However, the Ala55Val genotypes or alleles showed no statistical association with serum urate (TT+TC/CC: Beta = −0.070, *P* = 0.091; T/C: Beta = −0.047, *P* = 0.106) or hyperuricemia (TT+TC/CC: OR = 0.72, *P* = 0.173; T/C: OR = 0.72, *P* = 0.051). In the overweight subgroup, the genotypes of both loci were associated a lower serum urate (AA+AG/GG: Beta = −0.138, *P* = 0.001; TT+TC/CC: Beta = −0.130, *P* = 0.003) and a significant, or at least marginal, decreased risk of hyperuricemia (AA + AG/GG: OR = 0.62, *P* = 0.015; TT+TC/CC: OR = 0.74, *P* = 0.027). However, the alleles of the loci were associated with a lower serum urate level (A/G: Beta = −0.072, *P* = 0.019; T/C: Beta = −0.072, *P* = 0.019) but not with a decreased risk of hyperuricemia (A/G: OR = 0.75, *P* = 0.036; T/C: OR = 0.74, *P* = 0.027). Our results suggested a stronger effect of *UCP2* variants on overweight females (Table 2).

### Association between haplotypes and risk of hyperuricemia

As listed in Table 3, the haplotypes of the two loci were estimated in the total group of subjects and after stratification by gender. The −866G/A and Ala55Val variants were in strong linkage disequilibrium (D′ = 0.974, r^2^ = 0.936). The wild type haplotype G-C (−866G/A-Ala55Val) was applied as the reference one. Haplotype A-T made up for the most frequent one, while single mutation at −866G/A or Ala55Val each accounted for less than 1 percent (Table 3). In the total group of subjects, no haplotypes were correlated with susceptibility of hyperuricemia. In the female subgroups, haplotype A-T (−866G/A-Ala55Val) was associated with a decreased risk of hyperuricemia; however, this association was null in males. No further significant associations between hyperuricemia and other two rare haplotypes were found in our study, might partially due to the limited size of the rare haplotypes carriers (Table 3). These results correlated with the association between genotypes or alleles and hyperuricemia (Table 1).

## Discussion

Uncoupling protein 2 (UCP2) is present in the inner mitochondrial membrane and mainly decreases the ATP level and ROS produced by electron transport; therefore, UCP2 is involved in a board range of pathological processes. In the present study, we first focused on the relationship between *UCP2* variants and serum urate and hyperuricemia, potentially examining the scope of the loci related to hyperuricemia.

The present study revealed no association between the two polymorphisms of *UCP2* and serum urate or hyperuricemia in the total group of subjects. However, because serum urate is extensively influenced by gender differences, we stratified the total group of subjects and determined that −866G/A and Ala55Val were associated with serum urate and hyperuricemia in females^22^,^23^. Females with the −866G/A genotype AA+AG or allele A had lower serum urate and a decreased risk of hyperuricemia, indicating a protective role of −866G/A for hyperuricemia in females.

The −866G/A variant is a functional polymorphism located in the promoter region and putatively changes the transcription factor binding sites^7^. The wild type G allele in −866G/A was associated with lower *UCP2* mRNA expression^19^,^24^. Increased *UCP2* mRNA expression from the A allele was translated into an increased amount of UCP2 protein, with corresponding induced proton leak, decreased ATP/ADP ratio and enhanced elimination of ROS^10^,^19^. Hypermethylation in the promoter region could affect the binding of transcripation factors, causing aberrant gene expression. Consistent with our expectations, we found a typical CpG island in the *UCP2* promoter region, which included the locus of the −866G/A variant, using information from the University of California–Santa Cruz (UCSC; Santa Cruz, CA, USA) database (http://genome.ucsc.edu/cgi-bin/hgGateway). We believe the *UCP2* promoter variant −866G/A could shape this CpG island and protect the *UCP2* promoter region from DNA methylation, uncovering a novel underlying mechanism that determines −866G/A increases *UCP2* transcription.

Uric acid accumulation is caused by the acceleration of ATP degradation to AMP, a precursor of uric acid, and UCP2 could decrease the ATP level and lower redundant AMP for uric acid formation^7^,^25^. Moreover, an elevation of serum urate concentration occurs as a physiologic response to increased oxidative stress^26^. Because the ROS level could be down-regulated by UCP2, a counter-regulatory increase of serum urate as an antioxidant defense is less urgent. Therefore, the −866G/A variant in the promoter region might serve as a protective factor through a higher *UCP2* mRNA level and increased translation of the UCP2 protein, which might regulate ROS and modify the ATP/ADP ratio.

The other locus, Ala55Val, is a missense variant in exon 4 and is associated with an altered degree of uncoupling^7^. In our study, a protective effect for hyperuricemia and lower serum urate were observed in genotype TT and allele T in the female subgroups. However, the genetic effect of the Ala55Val variant was less clear. Several researchers identified an association of Ala55Val with the BMI level and type 2 diabetes mellitus (T2D), with controversial conclusions within cohorts, and few functional studies were performed^14^,^27^,^28^. Similar to −866G/A, the protective role of the Ala55Val variant for hyperuricemia might also be attributed to altered *UCP2* transcription.

In the male subgroups, a less statistically significant but possible effect of −866G/A and Ala55Val was observed for hyperuricemia risk and higher serum urate. Similar gender-associated genetic effects of *UCP2* variants were more or less observed for diseases other than hyperuricemia^7^. For example, Heidema *et al*. suggested a genetic effect of *UCP2* on weight gain was regulated through different mechanism in males and females^29^. Lee *et al*. demonstrated that the association between *UCP2* variants and BMI was more apparent among female subjects^30^. Cheurfa *et al*. confirmed the association of *UCP2* variants with coronary artery diseases in males but not females^31^. In the present study, we found *UCP2* variants −866G/A and Ala55Val had a stronger effect on females with hyperuricemia. One possible explanation for the gender-associated genetic effects of UCP2 might be a regulation role of sex hormones such as estrogen. Estrogen was reported to repress *UCP2* in a breast cancer cell line and papillary thyroid cancer cells^32^,^33^. Taken together, these results suggest the *UCP2* protein level was down-regulated by estrogen in females but reversed by the variants of −866G/A and Ala55Val, providing a plausible explanation for the specific protective effects of *UCP2* variants on females^32^.

Genetic effects on hyperuricemia and obesity have been widely recognized^3^. In the present study, we found that −866G/A and Ala55Val were associated with lower serum urate and a decreased risk of hyperuricemia in overweight females (Table 2). Among the normal weight females, the risk of hyperuricemia and serum urate level were associated with only −866G > A but not Ala55Val, while the association was observed with both the two loci in overweight group. These results indicated that obesity altered the genetic effects of the two loci on serum urate and overweight females might benefit more from the protective effects of *UCP2* variants. Among the two loci, the functional −866G > A promoter variant displayed a stronger genetic effect. The association analysis in underweight females might have limited statistic power due to the small sample size (cases/controls: 5/16) and a larger cohort would be necessary for further analysis. The interactions between obesity, uric acid and *UCP2* were complicated. BMI has long been viewed as an essential factor influencing uric acid^3^. *UCP2* transcription was activated by fatty acids^16^. A recent meta-analysis revealed that *UCP2* −866G/A and Ala55Val are associated with a risk of obesity^27^. Subtle intermediary obesity related phenotypes such as elevated triglycerides, total cholesterol concentrations, increased the risk of dyslipidemia and circulating leptin levels were also observed to be correlated with *UCP2* variants^34^. Based on these results, we assumed lipid metabolism material such as fatty acids participated in and enhanced the genetic effect of *UCP2* variants on serum urate regulation, explaining the stronger genetic effect of *UCP2* variants on females with higher BMI levels observed in the present study.

The −866G/A and Ala55Val variants were in strong linkage disequilibrium (D′ = 0.974, r^2^ = 0.936). The haplotype frequency analysis revealed that variants of the two loci were more in co-variant haplotype A-T (−866G/A-Ala55Val) compared with the single variant forms of G-T or A-C (less than 1%, Table 3). Haplotype A-T was associated with a decreased risk of hyperuricemia only in females, which was consistent with the genotype or alleles results. However, the small sample size of the two rare haplotypes might limited the power of association analysis with hyperuricemia risk to a certain extent. But we did observed a possible correlation between prevalence of hyperuricemia and the rare haplotype A-C even in such small sample size. A future larger cohort might provide further solid evidence for correlation between the two rare haplotypes and hyperuricemia.

## Conclusion

The present study identified a novel gene, *UCP2*, with two loci, −866G/A and Ala55Val; this gene influenced the serum urate concentrations and the risk of hyperuricemia in females. The associations of those loci were affected by gender and BMI. This study supported the potential involvement of this gene in the prevention, prediction and treatment of hyperuricemia.

## Materials and Methods

### Study design and approval statement

The study was approved by the Ethical Committees of the School of Life Science of Fudan University (approval number of 140). A total of 4332 subjects of similar lifestyle, diet, housing environment, and no medical treatment on serum urate level were enrolled from the Taizhou Longitudinal Study, a prospective cohort initiated to explore the environmental and genetic risk factors for common non-communicable diseases^35^. The use of the cohort was aimed to minimize the heterogeneity among subjects and to get the maximum robustness of the present study^36^. The written informed consent approved by the Ethical Committees of the School of Life Science of Fudan University was collected from all participates. The associations of common *UCP2* variants with serum urate and hyperuricemia were tested by linear regression and logistic regression with or without gender stratification, respectively. A body mass index (BMI) subgroup was also used for further analysis. All the experiment methods were performed in accordance with the approval document from Ethical Committees of the School of Life Science of Fudan University

### Participants

All subjects were enrolled from Taizhou Longitudinal Study^35^, of which 1387 individuals had serum urate level over 7 mg/dl and were treated as hyperuricemic patients, and 2945 individuals had normal serum urate (≤7 mg/dl) and were treated as healthy controls^37^. The subjects were divided into subgroups (underweight: BMI <18.5; normal weight: 18.50 ≤ BMI < 25; overweight: BMI ≥ 25) following the categories of the World Health Organization (WHO)^38^. The characteristics of the above individuals are shown in S.Table 1. Written informed consent was obtained from all subjects.

### Genetic analysis

Genetic analysis was carried out in accordance with approval document offered by the Ethical Committees of the School of Life Science of Fudan University. For genetic analysis, peripheral blood was collected from all the individuals with written informed consent included in this study. Genomic DNA was extracted from whole blood using the QIAamp DNA Blood Mini kit (QIAGEN, Germany) and was stored at −20 °C. The DNA concentration and quality (including optical density (OD) 260/280 and 260/230 measurements) were determined using a Nanodrop Lite spectrophotometer (Thermo Fisher Scientific, Waltham, MA, USA). Genotyping of −866G/A and Ala55Val in *UCP2* were performed by SNPscan according to the manufacturer’s instructions.

### Statistical analysis

The clinical characteristics were presented as the mean ± SD. Student’s t-test was used to test for a significant difference in the mean age, BMI and serum urate between hyperuricemia patients and healthy controls. The chi-square test was used to describe the gender distribution difference between hyperuricemia patients and healthy controls.

The chi-square test was used to test Hardy-Weinberg equilibrium (HWE) of the two loci. We conducted a logistic regression analysis to calculate adjusted odd ratio (OR) with 95% confidence interval (95% CI) and P-values to describe the distribution of −866G/A and Ala55Val adjusted for age and gender between hyperuricemia patients and healthy controls. A linear regression was performed to calculate Beta and *P*-values to estimate the effect on serum urate in different genotypes and alleles. Genotype GG, allele G of −866G/A and genotype CC, allele C of Ala55Val were used as references, respectively. Stratification into subgroups was performed on the basis of gender and different BMI values for further analysis. Haplotype frequencies between the hyperuricemic patients and controls were estimated by OR (95% CI) and chi-square test. The haplotype of the most frequent (−866G/A-Ala55Val, G-C) was used as the reference. A 2-sided *P*-value less than 0.025 was considered statistically significant after multiple correlation by Bonferroni method. The PHASE program (V2.1) was used for haplotype frequencies estimation, and SPSS 19.0 was used for the statistical analysis.

## Additional Information

**How to cite this article**: Yang, L. *et al*. Common *UCP2* variants contribute to serum urate concentrations and the risk of hyperuricemia. *Sci. Rep*. **6**, 27279; doi: 10.1038/srep27279 (2016).

## Supplementary Material

Supplementary Information

## Figures and Tables

**Table 1:** Association analysis of *UCP2* variants with serum urate and hyperuricemia.

SNP	Chr	Contig position	Function	A1^e^	A2	Gender	Genetic model	Case	Control	Serum urate		Hyperuricemia	
										Beta^a^	*P*-value^b^	OR (95% CI)^c^	*P*-value^d^
−866G/A	11	19458635	Promoter	G	A	Female	AA/GG	59/115	190/252	-0.078	0.015	0.66 (0.45, 0.96)	0.030
rs659366							AG/GG	153/115	425/252	-0.104	0.001	0.71 (0.53, 0.96)	0.025
							A/G	271/383	805/929	-0.054	0.006	0.80 (0.66, 0.96)	0.018
						Male	AA/GG	240/281	432/609	0.026	0.220	1.26 (1.01, 1.56)	0.038
							AG/GG	539/281	1035/609	0.020	0.328	1.16 (0.97, 1.38)	0.112
							A/G	1019/1101	1899/2253	0.016	0.209	1.12 (1.01, 1.25)	0.035
						Total	AA/GG	299/396	622/862	-0.008	0.644	1.05 (0.87, 1.26)	0.603
							AG/GG	692/396	1460/862	-0.012	0.474	1.03 (0.89, 1.20)	0.667
							A/G	1290/1484	2704/3184	-0.005	0.603	1.03 (0.94, 1.13)	0.554
Ala55Val	11	19452985	Missense	C	T	Female	TT/CC	57/111	199/240	-0.075	0.022	0.64 (0.43, 0.93)	0.020
rs660339							TC/CC	159/111	426/240	-0.082	0.012	0.77 (0.57, 1.04)	0.093
							T/C	273/381	824/906	-0.049	0.014	0.79 (0.66, 0.96)	0.016
						Male	TT/CC	241/277	434/602	0.020	0.328	1.23 (1.00, 1.53)	0.055
							TC/CC	541/277	1039/602	0.008	0.708	1.14 (0.95, 1.36)	0.164
							T/C	1023/1095	1907/2243	0.012	0.341	1.11 (1.00, 1.24)	0.053
						Total	TT/CC	298/388	633/842	-0.013	0.460	1.02 (0.85, 1.23)	0.824
							TC/CC	700/388	1566/842	-0.017	0.324	1.04 (0.89, 1.20)	0.652
							T/C	1296/1476	2731/3149	-0.008	0.421	1.01 (0.93, 1.11)	0.770

^a^Increase in serum urate associated with each copy of genotype or allele in −866G/A or Ala55Val.

^b^*P*-value for the multivariable linear regression analysis adjusting for age and gender.

^c^Increase in the odds of hyperuricemia with each copy of genotype or allele in −866G/A or Ala55Val.

^d^*P*-value for the logistic regression analysis adjusting for age and gender.

^e^Reference allele.

**Table 2:** Association of −866G/A and Ala55Val with serum urate and hyperuricemia in females with different BMI levels.

SNP	BMI level	Genetic Model	Case	Control	Serum urate		Hyperuricemia	
					Beta^a^	*P*-value^b^	OR^c^ (95% CI)	*P*-value^d^
**−866G/A**	**Underweight** BMI < 18.5	**AG/GG**	3/2	12/4	−0.118	0.600	0.78 (0.05, 12.55)	0.858
		**A/G**	3/7	12/20	−0.056	0.715	0.91 (0.17, 4.92)	0.916
	**Normal weight** 18.50 ≤ BMI < 25	**AA/GG**	15/35	107/128	−0.094	0.051	0.49 (0.25, 0.96)	0.038
		**AG/GG**	44/35	216/128	−0.100	0.039	0.70 (0.42, 1.17)	0.170
		**AA+AG/GG**	59/35	323/128	−0.095	0.022	0.65 (0.40, 1.05)	0.076
		**A/G**	74/114	430/472	−0.062	0.035	0.69 (0.50, 0.96)	0.029
	**Overweight** BMI ≥ 25	**AA/GG**	32/54	78/95	−0.102	0.042	0.61 (0.36, 1.03)	0.065
		**AG/GG**	76/64	129/95	−0.161	0.001	0.61 (0.40, 0.93)	0.022
		**AA+AG/GG**	108/64	257/95	−0.138	0.001	0.62 (0.42, 0.91)	0.015
		**A/G**	140/204	335/369	−0.072	0.019	0.75 (0.58, 0.98)	0.036
**Ala55Val**	**Underweight** BMI < 18.5	**TC/CC**	4/1	12/4	0.005	0.984	0.91 (0.05, 15.19)	0.947
		**T/C**	4/6	12/20	0.002	0.990	0.97 (0.18, 5.07)	0.969
	**Normal weight** 18.50 ≤ BMI < 25	**TT/CC**	15/33	107/128	−0.073	0.132	0.51 (0.26, 1.01)	0.054
		**TC/CC**	46/33	214/128	−0.072	0.140	0.78 (0.46, 1.30)	0.334
		**TT+TC/CC**	61/33	321/128	−0.070	0.091	0.72 (0.44, 1.16)	0.173
		**T/C**	71/112	438/470	−0.047	0.106	0.72 (0.52, 1.00)	0.051
	**Overweight** BMI ≥ 25	**TT/CC**	32/63	83/95	−0.106	0.036	0.58 (0.34, 0.98)	0.041
		**TC/CC**	77/63	174/95	−0.148	0.003	0.66 (0.43, 1.01)	0.056
		**TT+TC/CC**	109/63	257/95	−0.130	0.003	0.64 (0.44, 0.95)	0.027
		**T/C**	141/203	340/364	−0.072	0.019	0.74 (0.57, 0.97)	0.027

^a^Increase in serum urate associated with each copy of genotype or allele in −866G/A or Ala55Val.

^b^*P*-value for the multivariable linear regression analysis adjusting for age.

^c^Increase in the odds of hyperuricemia with each copy of genotype or allele in −866G/A or Ala55Val.

^d^*P*-value for the logistic regression analysis adjusting for age.

**Table 3:** The OR and 95% CI between estimated haplotype frequencies.

Gender	Haplotype^a^	Case	Control	OR (95%CI)	*P*-value
Total	A-T	1273 (46.9%)	2662 (45.3%)	1.06 (0.97–1.16)	0.241
	G-T	15 (0.6%)	38 (0.6%)	0.86 (0.45–1.64.)	0.767
	A-C	23 (0.8%)	68 (1.2%)	0.75 (0.44–1.25)	0.254
	G-C^b^	1403 (51.7%)	3112 (52. 9%)	1	
Male	A-T	1006 (48.13%)	1870 (45.77%)	1.07 (0.99–1.14)	0.080
	G-T	17 (1.54%)	35 (1.55%)	0.99 (0.67–1.47)	1.000
	A-C	11 (1.00%)	27 (1.20%)	0.88 (0.53–1.45)	0.729
	G-C^b^	1084 (51.87%)	2216 (54.23%)	1	
Female	A-T	267 (40.8%)	792 (45.8%)	0.80 (0.66–0.97)	0.017
	G-T	4 (0.6%)	11 (0.6%)	0.86 (0.19–2.93)	1.000
	A-C	6 (0.9%)	32 (1.8%)	0.44 (0.15–1.09)	0.070
	G-C^b^	337 (57.6%)	895 (53.05%)	1	

^a^Haplotype: −866G/A-Ala55Val.

^b^Haplotype G-C, which has the highest frequency, as a reference.
