# Supplementary material for: Common UCP2 variants contribute to serum urate concentrations and the risk of hyperuricemia
Source: Sci Rep. 2016 Jun 8;6:27279. doi: 10.1038/srep27279 (PMC4897637; doi:10.1038/srep27279)
Supplement: Supplementary Information [file srep27279-s1.pdf]

Common *UCP2* variants contribute to serum urate concentrations and the risk of hyperuricemia

Luyu Yang[1¶], Zheng Dong[1¶], Jingru zhou[1], Yanyun Ma[1], Weilin Pu[1], Dongbao Zhao[2], Hongjun He[3], Hengdong Ji[4], Yajun Yang[1,5], Xiaofeng Wang[1,5], Xia Xu[2], Yafei Pang[2], Hejian Zou[6,7], Li Jin[1,5], Chengde Yang[8*], Jiucun Wang[1,5,7*]

[1]State Key Laboratory of Genetic Engineering and Ministry of Education Key Laboratory of Contemporary Anthropology, Collaborative Innovation Center for Genetics and Development, School of Life Sciences, Fudan University, Shanghai, China.

[2] Division of Rheumatology and Immunology, Changhai Hospital, Shanghai, China.

[3] Division of Rheumatology, Taixing People's Hospital, Jiangsu Province, China.

[4] Division of Rheumatology, Taizhou People's Hospital, Jiangsu Province, China.

[5] Fudan-Taizhou Institute of Health Sciences, Taizhou, Jiangsu Province, China.

[6] Division of Rheumatology, Huashan Hospital, Fudan University, Shanghai, China.

[7] Institute of Rheumatology, Immunology and Allergy, Fudan University, Shanghai, China.

[8]Division of Rheumatology, Ruijin Hospital, Shanghai Jiaotong University School of Medicine, Shanghai, China.

*Corresponding author

E-mail: yangchengde@sina.com (Chengde Yang), jcwang@fudan.edu.cn (Jiucun Wang)

[¶]These authors equally contributed to this study.

S.Table 1. Characteristics of hyperuricemia and healthy control*

	Control	Hyperuricemia
Number	2945	1387
Male/Female	2077/868	1060/327
Age (year)	69.00±9.25	67.91±11.93
Height (cm)	161.37±7.52	162.63±7.54
Weight (kg)	63.71±9.96	68.56±11.12
BMI	24.43±3.24	25.88±3.52
Smoking	87.05%	72.33%
Serum Urate (umol/L)	300.73±63.13	482.30±61.14

*All data shown in mean±SD
